# Detection of Cleaved Stx2a in the Blood of STEC-Infected Patients

**DOI:** 10.3390/toxins15120690

**Published:** 2023-12-08

**Authors:** Elisa Varrone, Domenica Carnicelli, Xiaohua He, Marco Grasse, Karin Stampfer, Silke Huber, Sára Kellnerová, Pier Luigi Tazzari, Francesca Ricci, Paola Paterini, Gianluigi Ardissino, Stefano Morabito, Dorothea Orth-Höller, Reinhard Würzner, Maurizio Brigotti

**Affiliations:** 1 Department of Medical and Surgical Sciences (DIMEC), University of Bologna, Via San Giacomo 14, 40126 Bologna, Italy; elisa.varrone2@unibo.it (E.V.); domenica.carnicelli@unibo.it (D.C.); paola.paterini@unibo.it (P.P.); 2 Western Regional Research Center, U.S. Department of Agriculture, Agricultural Research Service, 800 Buchanan Street, Albany, CA 94710, USA; xiaohua.he@usda.gov; 3 Institute of Hygiene and Medical Microbiology, Medical University of Innsbruck, 6020 Innsbruck, Austria; marco.grasse@i-med.ac.at (M.G.); karin.stampfer@i-med.ac.at (K.S.); silke.huber@i-med.ac.at (S.H.); sara.kellnerova@i-med.ac.at (S.K.); reinhard.wuerzner@i-med.ac.at (R.W.); 4 Immunohematology and Transfusion Center, S. Orsola-Malpighi Hospital, Via Massarenti 9, 40138 Bologna, Italy; pierluigi.tazzari@aosp.bo.it (P.L.T.); francesca.ricci@aosp.bo.it (F.R.); 5 Center for Applied Biomedical Research-CRBA, University of Bologna, IRCCS Azienda Ospedaliero-Universitaria di Bologna, 40138 Bologna, Italy; 6 Center for HUS Control, Prevention and Management, Fondazione IRCCS Ca’ Granda Ospedale Maggiore Policlinico, Via Commenda 9, 20122 Milano, Italy; ardissino@italkid.org; 7 European Reference Laboratory for *Escherichia coli*, Istituto Superiore di Sanità, 00161 Rome, Italy; stefano.morabito@iss.it; 8 MB-LAB—Clinical Microbiology Laboratory, 6020 Innsbruck, Austria; do@mb-lab.com

**Keywords:** hemolytic uremic syndrome, cleaved Shiga toxin 2a, Shiga toxin-producing *Escherichia coli*

## Abstract

Typical hemolytic uremic syndrome (HUS) is mainly caused by Shiga toxin-producing *Escherichia coli* (STEC) releasing Shiga toxin 2 (Stx2). Two different structures of this AB5 toxin have been described: uncleaved, with intact B and A chains, and cleaved, with intact B and a nicked A chain consisting of two fragments, A1 and A2, connected by a disulfide bond. Despite having the same toxic effect on sensitive cells, the two forms differ in their binding properties for circulating cells, serum components and complement factors, thus contributing to the pathogenesis of HUS differently. The outcome of STEC infections and the development of HUS could be influenced by the relative amounts of uncleaved or cleaved Stx2 circulating in patients’ blood. Cleaved Stx2 was identified and quantified for the first time in four out of eight STEC-infected patients’ sera by a method based on the inhibition of cell-free translation. Cleaved Stx2 was present in the sera of patients with toxins bound to neutrophils and in two out of three patients developing HUS, suggesting its involvement in HUS pathogenesis, although in association with other bacterial or host factors.

## 1. Introduction

Typical hemolytic uremic syndrome (HUS) is a thrombotic microangiopathy, presenting with hemolytic anemia, thrombocytopenia, and acute renal failure, which occurs as a severe sequela of Shiga toxin-producing *Escherichia coli* (STEC) gastrointestinal infections [1,2,3,4]. These pathogenic bacteria release potent exotoxins called Shiga toxins (Stx) as major virulence factors [1,2,3,4]. The syndrome is also termed eHUS for enterohemorrhagic *E. coli* associated HUS.

During the pathogenesis of HUS, before the toxins act on the target endothelial cells of the kidney, several Stx forms are transported in the bloodstream: (i) soluble Stx [5,6]; (ii) Stx bound to circulating cells (neutrophils, monocytes, erythrocytes and platelets) exposing the receptors globotriaosylceramide (Gb3Cer) [7,8,9,10] and Toll-like receptor 4 (TLR4) [1,5], eventually inducing the formation of leukocyte–platelet aggregates and pathogenic extracellular vesicles; and (iii) Stx associated with blood-cell-derived microvesicles [5,10]. The latter form is considered the main factor for the transition from bloody diarrhea to HUS in approximately 15% of STEC-infected patients, especially children under 3 years, with a mortality rate of 3–5% [1,3,10,11,12]. These extracellular vesicles can attack renal target cells by delivering Stx and other pathogenic factors, such as tissue factor and/or activated complement components which concur with HUS pathogenesis [13,14,15,16,17].

**Figure 1:**
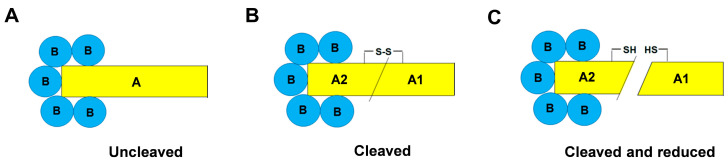
Structure of Stx2. (**A**) Native form composed of an A chain non-covalently bound to five identical B chains; (**B**) cleaved form composed of A1 and A2 fragments linked by a disulfide bound. The A2 fragment is connected to the B pentameric ring. (**C**) Cleaved and reduced form.

Stx are a family of AB5 bacterial toxins consisting of two main types, Stx1 and Stx2, and several subtypes in each type [18]. Stx2a is the major subtype associated with the development of HUS in humans [19]. Stx consists of a single 32 kDa-A chain non-covalently bound to five identical B chains (7.7 kDa each), forming a pentameric ring (uncleaved Stx, Figure 1A) [1,20]. The A chain is a proenzyme that is enzymatically cleaved at arginine residues 247/248 or 250/251 (Stx2/Stx1) by proteases resulting in two fragments linked by a disulfide bond: A1, the 27.5 kDa enzymatically active fragment, and the 4.5 kDa A2 fragment connected to the B pentameric ring (cleaved Stx, Figure 1B) [21,22]. Under reducing conditions, the disulfide bond is broken, usually within cells, permitting the enzymatically active A1 fragment to express its deadenylating activity on 28S rRNA in ribosomes, resulting in a decreased ribosomal affinity for eukaryotic elongation factors (eEF1 and eEF2) and leading to an irreversible arrest of translation [1,2,21,22,23]. Another intracellular target of Stx is DNA in chromatin that is serially deadenylated, leading to the formation of nuclear apurinic sites [1,2,10]. In target cells, the proteolytic cleavage of the A chain can occur during the retrograde transport of the toxin, by the protease furin, which recognizes the sensitive region (Arg247/248-X-X-Arg250/251), which is the consensus motif also recognized by trypsin [22]. However, a cleavage activity, although different, has also been found in the intestinal mucus [24,25] or can be induced by bacterial proteases released after the lysis of bacteria [1,2].

Both cleaved unreduced Stx2a, which has a single nick in the A chain, resulting in fragments A1 and A2 being connected by a disulfide bridge, and uncleaved Stx2a (Figure 1) have recently been found to be biologically active as they intoxicate human cells expressing Gb3Cer (Vero and Raji cells) similarly, but functionally differently [26]. Indeed, they have different binding properties for circulating cells and host serum components as well as differing in the formation of leukocyte/platelet aggregates [26]. Cleaved Stx does not bind to human neutrophils via TLR4 or to the same receptor present on monocytes and platelets, while it is capable of binding to complement factor H; uncleaved toxins show contrary features and stimulate the formation of aggregates between leukocytes and platelets [26]. The lack of interaction of the cleaved form of Stx with human neutrophils suggests that the binding site for neutrophils is probably very close to the A subunit cleavage site but does not correspond to the active site of the toxin. In addition, the formation of neutrophil/platelet and monocyte/platelet aggregates induced by the uncleaved form indicates that TLR4 has an important role in this process, given that all the involved cells express this receptor. Concerning the human serum amyloid P component (HuSAP), cleaved and uncleaved Stx2 seem to have the same behaviors. Indeed, both forms interact with this blood protein [26], which prevents their binding to Gb3Cer receptor [27,28,29] and promotes their interaction with TLR4 [1,26].

To sum up, these observations suggest that Stx must be uncleaved to bind human neutrophils, since these cells do not express the Gb3Cer receptor. The other circulating cells involved in toxin binding (platelets and monocytes) can interact with both forms, as they express both Gb3Cer and TLR4. These multiple blood interactions, which markedly differ depending on the toxin form (uncleaved or cleaved), are relevant to the pathogenesis of HUS.

Although the structure, the mechanism of action and the different properties of the two forms of the toxin are well known, whether the A subunit is still intact or already cleaved when the holotoxins enter the bloodstream and challenge the human blood components is unclear. In addition, it remains to be established whether the cleaved form of Stx2a can be found in the blood of STEC-infected patients. The outcome of STEC infections and the consequent onset of HUS could therefore be influenced by the percentages of uncleaved or cleaved toxins circulating in patients’ blood, since the two forms of toxin could produce different amounts of leukocyte/platelet aggregates and, likely, different amounts of pathogenic microvesicles. Thus, the characterization of the structure of the toxins present in the blood of patients could be useful for a better understanding of the mechanisms underlying the pathogenesis of HUS.

## 2. Results

### 2.1. The Detection Tool: A Luminometric Cell-Free Translation System (LCFTS)

**Figure 2:**
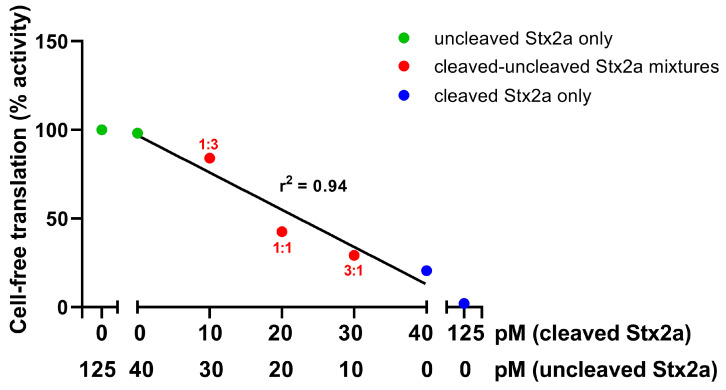
Different mixtures containing trypsin-cleaved and uncleaved Stx2a (red circles) accounting for different ratios of the two forms of the toxin were added to LCFTS at constant Stx2 concentration (40 pM). The ratio of cleaved/uncleaved Stx2a for each mixture is also reported (red numbers). Uncleaved Stx2a alone (green circles) and cleaved Stx2a alone (blu circles) were also added for comparison. Percentage activities of cell-free translation (y-axis) are plotted against uncleaved Stx2a concentrations and cleaved Stx2a concentrations, both shown on the x-axis. Addition of samples containing the reagents used in the Stx2a cleaving procedure (trypsin and PMSF) at the same concentrations found in the assayed cleaved Stx2 samples had no effect on translation.

To detect the cleaved form of Stx2a in STEC-infected patients’ sera, a luminometric cell-free translation system (LCFTS) was used. The assay was based on rabbit reticulocyte lysate reconstituted with human ribosomes that translate added synthetic mRNAs coding for luciferase [30]. In this system, the two forms of Stx2a (trypsin-cleaved and uncleaved) showed different behaviors when a reducing agent (DTT) was added, i.e., under these conditions the trypsin-cleaved Stx2a boosted its activity, whereas the uncleaved Stx2a activity was not affected. Indeed, the high concentrations of DTT in the assay break up the disulfide bridge between the two fragments of cleaved toxin (Figure 1C), allowing the A1 fragment which strongly inhibits protein synthesis to be released. The concentrations of trypsin-cleaved toxin measured by this method were very similar to those determined in patients by ELISA assays (2–6 ng/mL, approximately 30–90 pM) [30]. At those concentrations, uncleaved Stx2a had no effect on the translation system. The behavior of the system is exemplified by the experiments depicted in Figure 2: the addition of mixtures of uncleaved and trypsin-cleaved Stx2a (Figure 2, red circles) to the system at a constant total Stx2a concentration (40 pM) induced translation inhibitions that are dependent on the concentration of cleaved Stx2a only.

### 2.2. Detection of the Cleaved form of Stx2a in STEC-Infected Patients’ Sera by LCFTS

As shown in Table 1, sera from eight STEC-infected patients were analyzed for the determination of the cleaved form of the toxin using LCFTS. The diagnosis of STEC infection was confirmed by means of the identification of the gene encoding Stx2 in fecal extracts, by isolation of the STEC strain from the stool samples or by the detection of serum Stx2 by ELISA. The presence of cleaved Stx2a was observed in four out of eight sera, and its concentration was calculated as described in [30] and compared to that obtained by ELISA. 

**Figure 3:**
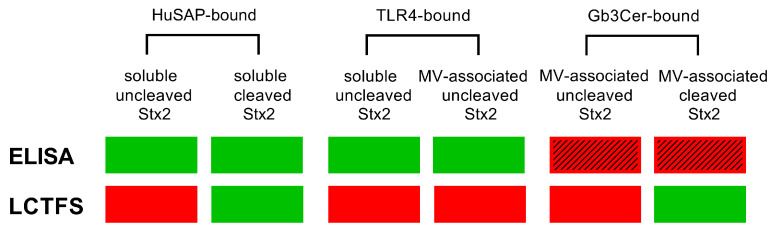
Different forms of Stx2a bound to different receptors/serum factors are recognized by ELISA or by the luminometric cell-free translation system (LCFTS) as described in Materials and Methods. The diagram shows different toxin forms based on the bound molecule (HuSAP, TLR4 or Gb3Cer), the soluble (bound to serum factors) or particulated (microvesicle (MV)-associated) condition and the status (cleaved or uncleaved) of the A chain. Each specific toxin form is either detected (green) or not detected (red) by the specific assay (ELISA, LCFTS), as experimentally demonstrated (filled rectangles) or as inferred according to its features (red rectangles with step lines).

**Figure 4:**
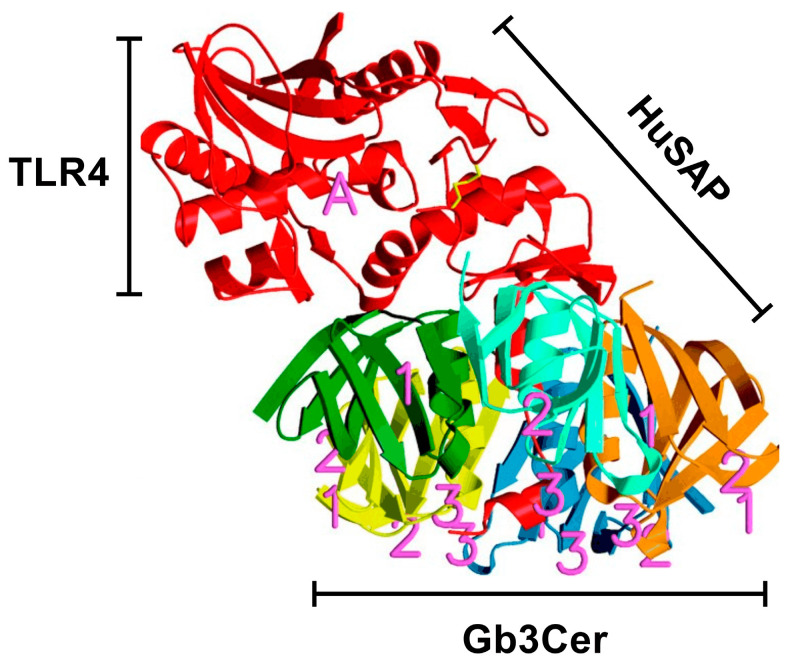
Ribbon diagram of Stx2 reproduced with permission from Fraser et al. [31] with modifications. The five B-subunits are orange, cyan, green, yellow and blue; putative Gb3Cer-binding sites on the B-pentamer are marked with magenta numbers. The A subunit is red, with the active site corresponding to magenta letter A. The cysteine residues forming the bond between A1 and A2 fragments are depicted in yellow. The binding site for TLR4 is located in the A1 fragment, whereas HuSAP interacts with both subunits, although the specific amino acids of the toxin involved in such bindings have not been determined.

The concentration of cleaved toxin, calculated according to LCFTS, was lower than the total concentration detected by ELISA in two out of four cases while in the remaining two patients the observed concentration of the cleaved toxin was higher. This could be explained by considering the different features of the two methods (Figure 3) and that the B-pentamer of Stx2 binds to Gb3Cer [31], the A chain is recognized by TLR4 [10] and HuSAP interacts with both subunits [29], as shown in Figure 4.

The toxin is recognized both in uncleaved and cleaved form by ELISA; however, the capturing antibody binds to the B pentamer, and consequently the soluble toxin is identified, as well as those toxin molecules interacting with TLR4 on the surface of microvescicles. In contrast, it is conceivable that the toxin bound to microvesicles through Gb3Cer/B chains interactions (Figure 4) is not recognized by the B-chain-interacting capturing antibody of the ELISA (Figure 3).

It is worth noting that Stx2a bound to Gb3Cer could be cleaved or uncleaved (Figure 3). By using LCFTS, when the toxin is bound via the B chain pentamer to the Gb3Cer expressed by microvesicles of platelet or monocyte origin, it exposes the A subunit which, if present in cleaved form, is documented by the system, similarly to soluble cleaved Stx2a. On the other hand, Stx2a bound to TLR4 via its A subunit is always uncleaved. For these reasons, the concentration of cleaved toxin in some patients turns out to be greater than the concentration of toxin detected by ELISA, i.e., the latter assay could underestimate the total Stx2a amount due to the lack of detection of the Gb3Cer-bound cleaved toxin (Figure 3). In conclusion, given the different forms of the toxin and the extreme variety of toxin-binding factors in blood (Figure 3 and Figure 4), the determinations obtained by ELISA or LCFTS only approach the total amount of Stx2a. For this reason, the combined information obtained by ELISA and LCFTS provides the best picture. 

**Figure 5:**
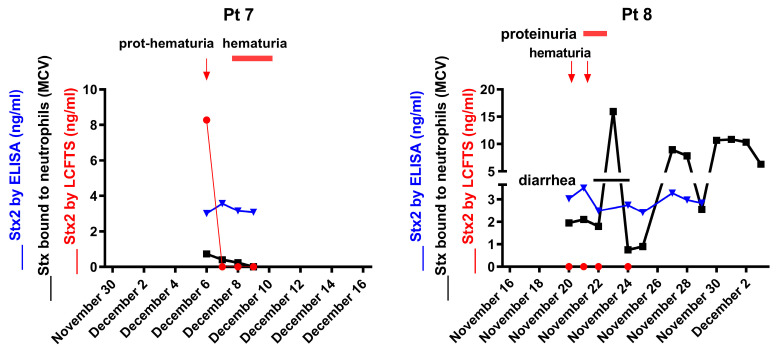
Time-course analysis of cleaved Stx2 detection in two STEC-infected patients. Stx2 on neutrophils (black) was detected by indirect flow cytometric analysis, the amount of Stx2 (blue) in patients’ sera was measured by ELISA and the amount of cleaved Stx2 (red) was measured by LCFTS. The presence of clinical manifestations such as diarrhea (solid horizontal line) and proteinuria/hematuria (red horizontal lines or red arrows) was also shown. Numbers at the top of each panel identify the patient listed in Table 1.

A time-course analysis of cleaved Stx2 detection was performed in two patients (Figure 5) when they were symptomatic. The results showed a lack of detection over time in patient 8 and an early one-day peak of cleaved Stx2 followed by a lack of detection for 3 days in patient 7. These results suggest that the proper detection of cleaved Stx2 would require early and repeated determinations in each patient. Unfortunately, time-course analyses were not performed in patients 1-6 due to low amounts of sera.

## 3. Discussion

The outcome of STEC infections and the onset of HUS could be influenced by the percentages of toxins present in uncleaved or cleaved form circulating in the patient’s blood, as in other toxin-mediated diseases. *Bacillus anthracis* protective antigen and toxin A from *Clostridium difficile* changed their properties and their contributions to disease development after proteolytic cleavage, as in the case of Stx2a [32,33].

By using the newly developed method, LCFTS, we identified the cleaved form of Stx2a in 50% of eight STEC-infected patients’ sera. The cleaved form of the toxin was present in two out the three patients who developed HUS and in two out of five who recovered, suggesting that there is not a clear correlation between the presence of this form of the toxin in serum and the onset of HUS. However, cleaved Stx2a may act in cooperation with other pathogenic factors (including uncleaved Stx2a), so that the relative amounts of the different factors, rather than their mere presence, may play a role in triggering HUS or in modulating the severity of symptoms. Regardless of any speculation, since the cleaved form of the toxin has only been characterized in vitro, its detection in the blood of STEC-infected patients seems to be a relevant finding which may open perspectives for more extensive studies. 

Bloody diarrhea, watery diarrhea, hematuria and proteinuria were found both in the patient group with the cleaved form of the toxin and in the patient group in which it was not detected. A common feature of all the patients with the cleaved form of Stx2a was the presence of the neutrophil-bound toxin (Table 1). Since the binding of Stx2a to neutrophils is only possible if the toxin is not cleaved, the presence of the cleaved form of Stx2a in patients with neutrophil-bound Stx2 suggests two hypotheses: (i) the toxin enters into the circulation from the gut uncleaved, at least in part, in order to bind to neutrophils, and/or (ii) it is cleaved after binding to neutrophils.

Another hypothesis is that part of the toxin may be cleaved in an earlier phase by some bacterial proteases and is released into the bloodstream already in the cleaved form. In this case, it can only recognize the Gb3Cer receptor expressed by circulating cells and therefore only binds to platelets and monocytes even though, given its properties, it stimulates the formation of leukocyte–platelet aggregates to a lesser extent. Alternatively, since microvesicles have the same features as the cells from which they derive, those formed by monocytes and platelets can have Stx2a bound to Gb3Cer via the B chain pentamer, leaving the toxin A chain exposed and available for subsequent cleavage by host serum proteases. Finally, host intestinal proteases could also be responsible for the cleavage of toxins before their release in blood. Recently, two serine proteases, trypsin and chymotrypsin-like elastase 3B, have been identified as possible candidates that can induce the cleavage of Stx2a in the gut [34]. Given the complexity of these scenarios, the identification and the source of protease/s inducing the toxin cleavage needs further investigation.

## 4. Conclusions

The application of LCFTS has proved to be a useful tool for studying and obtaining more information on the presence of the cleaved form of Stx2a in patients and on the possible relationship with clinical symptoms. However, whether it contributes to HUS onset and to what extent remains to be clarified. To better understand the role of the cleaved form of Stx2a in the pathogenesis of HUS, it will be necessary to perform targeted kinetic studies with repeated determinations over time to investigate the evolution of the clinical symptoms in STEC-infected patients and, at the same time, the appearance of cleaved Stx2a in sera.

## 5. Materials and Methods

### 5.1. Stx2a Purification

Stx2a was produced from strain C600ϕ933W, an *E. coli* K12 strain containing the bacteriophage carrying the Stx2a gene of the STEC EDL 933 strain, which was supplied by Dr. Alison O’Brien (Department of Microbiology and Immunology, Uniformed Services University of the Health Sciences, Bethesda, MD, USA), and isolated according to [35]. Briefly, the toxin was purified starting from the ammonium sulfate-precipitated proteins of the culture supernatant by passage through a receptor analogue affinity column (1.5 cm × 0.6 cm, 1 mL total volume) in which bovine serum albumin (BSA) linked to a terminal galabiose (Galα1-4Galβ-*O*-spacer—BSA, Glycorex, Lund, Sweden) was coupled to cyanogen bromide-activated Sepharose. To remove any trace of contaminant endotoxin, Stx2a was applied to ActiClean Etox columns (Sterogene Bioseparations, Carlsbad, CA, USA). The Stx2a preparation was quantified by a Lowry assay and assayed using Limulus Pyrogen Plus amebocyte lysate (Cambrex, Walkersville, MD, USA), demonstrating the presence of a low amount of LPS (0.73 ng/mg Stx2a).

### 5.2. Cleavage of Stx2a

To obtain the cleaved form of Stx2a, the toxin (4 μg) was treated with 50 ng of trypsin (1 mg/mL in 0.1 mM HCl, diluted to 0.05 ng/mL with PBS) in 10 μL PBS pH 7.5 and incubated for 1 h at 37 °C. Then, to inactivate trypsin, 0.7 ng of the trypsin inhibitor phenylmethylsulfonyl fluoride (PMSF, 1 mg/mL dissolved in absolute ethanol and diluted to 0.7 μg/mL with water) was added and incubated for 10 min at 37 °C [26,30].

### 5.3. Human Samples from STEC-Infected Patients

Serum samples from 8 STEC-infected patients (5 males and 3 females, median age 5.7 years), 3 with overt HUS, were collected and stored at −20 °C. Parents of the children gave their informed consent for inclusion before they participated in the study. The study was conducted in accordance with the Declaration of Helsinki, and the protocol was approved by the Ethics Committee of the Fondazione IRCCS Ca’ Granda Ospedale Maggiore Policlinico, Milan, Italy (18 May 2010). The detection of Stx genes in enrichment cultures of feces using real-time PCR and the isolation of STEC strains and the search for antibodies to lipopolysaccharide (LPS) were performed as described in [36,37,38,39]. Stx bound to neutrophils was detected by indirect flow cytometric analysis in the presence of monoclonal antibodies to Stx1 and Stx2 (Stx1–13C4, Stx2-BB12; Toxin Technology, Sarasota, FL, USA) [5]. 

Stx2 present in patients’ sera was quantified by a specific improved enzyme-linked immunosorbent assay (ELISA), as previously reported [40]. Briefly, the ELISA-based method was specifically designed for the detection of Stx2 in serum samples. Since this toxin interacts with several blood factors, including HuSAP, a preincubation step of sera with the chaotropic agent guanidinium chloride (200 mM) was performed to dissociate the toxin from the bound molecules. Subsequently, monoclonal (Stx2-2) and polyclonal (Stx2-pAb) antibodies against Stx2 were used in the ELISA. Each sample was run in duplicate. Patients’ sera were also used for the detection of the cleaved form of Stx2a. Control serum samples were obtained from three adults and used in the study after obtaining informed consent.

### 5.4. Detection of the Cleaved Form of Stx2a in Serum Samples from STEC-Infected Children by LCFTS

The detection of the cleaved form of the toxin was performed according to a published methodological paper [30]. Briefly, human serum samples were treated with immobilized protein G to remove an endogenous cell-free translation inhibitor, and 5-fold concentrated by centrifugation on Centricon 30 [30]. Then, the obtained samples were applied to a luminometric acellular translation system derived from rabbit reticulocyte lysate, fractionated, and reconstituted with human ribosomes that translate synthetic exogenous mRNA encoding the enzyme luciferase (*Renilla reniformis*) [30]. Each experiment was performed in duplicate. The system is able to discriminate the two forms of Stx2a based on their different inhibitory effects on protein synthesis under reducing conditions (80 mM DTT), since the IC_50_ values are in the picomolar range for the trypsin-cleaved toxin and in the nanomolar range for the uncleaved toxin [30].

## Figures and Tables

**Table 1:** Detection of cleaved Stx2a in STEC infected patients’ sera.

Pt *	Sex	Age (y)	Clinical Symptoms	*E. coli* Serogroup	Toxin Type	RT-PCR Stx2 Gene	Stx2 by ELISA (ng/mL)	Cleaved Stx2 by LCFTS (ng/mL)	Neutrophil- Bound Stx2
1	f	1.7	BD, D, H, P, HUS	O26	Stx2	+	3.36	2.90	+
2	f	5.6	BD, D, P, HUS	O157	Stx2	+	3.37	11.45	+
3	m	8.3	BD, H, P, HUS	O127	Stx2	+	2.41	-	+
4	f	10.2	BD, D, P	O145	Stx2	−	2.23	1.98	+
5	m	10.2	BD	O8	Stx2	+	2.38	-	−
6	m	3.3	BD, D, P	nd	Stx2	−	2.13	-	−
7	m	6.4	H, P	O127	Stx2	+	3.00	8.27	+
8	m	5.7	D, H, P	O127	Stx2	+	3.02	-	+

* Clinical and microbiological features of 8 STEC-infected patients: sex (3 females and 5 males); age (median 5.7 years, range 1.7–10.2 years); clinical symptoms as bloody diarrhea (BD, 6/8) and/or diarrhea (D, 5/8), hematuria (H, 4/8), proteinuria (P, 7/8), hemolytic uremic syndrome (HUS, 3/8); *E. coli* serogroup determination; toxin type according to the presence of the gene encoding Stx2 in fecal extracts identified by real time-polymerase chain reaction (RT-PCR) or by direct identification of the toxin type in serum by enzyme-linked immunosorbent assay (ELISA, ng/mL); cleaved Stx2 identified by luminometric cell-free translation system (LCFTS, ng/mL); neutrophil-bound Stx2 (6/8).

## Data Availability

The data presented in this study are available on request from the corresponding author.
